# When a Painful Rash Keeps Recurring: A Case of Seronegative Amyopathic Dermatomyositis Without Neurological Sequelae

**DOI:** 10.7759/cureus.42727

**Published:** 2023-07-31

**Authors:** Ahmed Harazeen, Brian Walter, Xiangping Li, Chilvana Patel

**Affiliations:** 1 Neurology, University of Texas Medical Branch, Galveston, USA; 2 Neurology, Houston Methodist Hospital, Houston, USA

**Keywords:** painful rash, seronegative amyopathic dermatomyositis, amyopathic dermatomyositis, dermatomyositis, inflammatory myositis

## Abstract

We present a case of seronegative amyopathic dermatomyositis (SADM). This clinical entity should be considered in the differential diagnosis of patients with recurring, painful erythematous skin manifestations, and requires close monitoring for the development of neurological manifestations and malignancy. SADM is a rare autoimmune disease that affects the skin and muscles. It is considered a subtype of dermatomyositis (DM), which is a systemic autoimmune disease. The exact cause of SADM is not fully understood but is believed to involve a complex interplay between genetic, environmental, and immunological factors. The diagnosis of SADM is typically made based on clinical evaluation, blood tests, muscle biopsy, and skin biopsy. Treatment options for SADM may include corticosteroids, immunosuppressive drugs, and other supportive measures to manage symptoms and prevent disease progression. A 30-year-old female presented with symptoms of intermittent burning, painful rash primarily on the hands and face. Her medical history was remarkable for a six-year history of multifocal joint pain, chronic low back pain, and intermittent, painful recurring rash in the upper body (face, neck, and chest). Neurological examination revealed scalp tenderness and arthralgia in the upper extremities, with normal motor strength examination. Skin findings included described an erythematous rash on the arms and hands bilaterally. Skin punch biopsy showed compact orthokeratosis, atrophy of the epidermis, interface changes, and increased dermal mucin on the colloidal iron stain, which are suggestive of DM. Electromyography and nerve conduction study were normal. The MRI of the left thigh was normal. C3 and C4 levels were reduced. The extended muscle-specific myositis panel including MDA5 was negative. The patient was placed on a multidrug regimen, including methotrexate, hydroxychloroquine, and prednisone. Within one year of follow-up, she was found to have reductions in skin manifestation and flare-ups. Clinicians should consider amyopathic DM (ADM) in the differential diagnosis of patients with recurring, painful skin manifestations. This condition can be easily overlooked as the development of neurological sequelae may be present much later in the course. We highlight the need for a multi-disciplinary management approach for patients with this unique diagnosis. Close monitoring for the development of neurological manifestations and associated sequelae including malignancy is recommended.

## Introduction

Dermatomyositis (DM) is a rare, immune-mediated inflammatory disease characterized by muscle weakness and skin manifestations [1]. Symmetric proximal muscle weakness is a hallmark feature of DM [2]. Symptoms typically present in a subacute fashion, with skin manifestations occurring either concomitantly or preceding the onset of muscle weakness. Skin manifestations present in a variety of forms, with rash commonly observed in photosensitive areas. Common skin manifestations include heliotrope rash, which affects the upper eyelids, rash over extensor joint surfaces such as the elbows and knees, V sign on the chest, Shawl sign, and Gottron sign [2]. DM is frequently accompanied by extra-muscular manifestations affecting the cardiac, pulmonary, gastrointestinal, and joint systems. Muscle-related enzymes, including creatine kinase (CK), aldolase, lactate dehydrogenase, and aspartate aminotransferase, are typically elevated [1]. CK levels may range from normal to significantly elevated up to 50,000 IU/L. Histopathological examination reveals perifascicular muscle fiber atrophy, inflammatory infiltrates, and vasculopathic changes involving capillary degradation, with CD4+ predominant lymphocytes in perimysium and perivascular regions. MRI of muscles may show variable findings, including muscle atrophy, edema, fasciitis, and fatty replacement [3].

Bohan and Peter's diagnostic criteria for DM include the presence of at least four out of five cardinal features, including symmetric proximal muscle weakness, elevated serum muscle enzymes, characteristic electromyography changes, typical skin lesions such as heliotrope rash, and characteristic muscle biopsy changes [4]. The European League Against Rheumatism and the American College of Rheumatology established new classification criteria for idiopathic inflammatory myopathies in 2017, which include age of onset, pattern of muscle involvement, presence of skin manifestations, and laboratory findings [5]. Clinical subtypes of DM include juvenile DM, clinically amyopathic DM (CADM), and seronegative amyopathic DM (SADM). Juvenile DM is defined as onset before the age of 18 and typically presents with more gastrointestinal complications and increased development of muscle and skin calcifications [6].

CADM is characterized by skin manifestations but without muscle weakness and is classified as either hypomyopathic or amyopathic DM (ADM) [3]. Hypomyopathic DM does not present with characteristic muscle weakness, but other abnormalities can be identified through biopsy, EMG testing, or laboratory studies [5]. The prevalence rate of DM is 9.63 per 1 million people, and CADM has a prevalence rate of 2.08 per 1 million people [7]. However, there is limited research examining CADM prevalence across larger, more diverse populations, and thus prevalence rates are not well-established [1]. DM is more commonly observed in women, with a 2:1 female-to-male ratio, and is seen most frequently in African Americans [1]. There are five well-known myositis-specific antibodies (MSA) associated with DM: anti-Mi2, anti-TIF-1y, anti-NXP2, anti-MDA5, and anti-SAE. MSA phenotypes have varying risks and severities of the disease and, thus, provide clinical guidance and prognostic capability in managing these patients [8]. Anti-MDA5 has been found to be associated with CADM and the development of progressive interstitial lung disease (ILD). Adult anti-MDA-5-positive patients often lack the classic DM findings of perifascicular muscle atrophy and capillary loss on muscle biopsy [8].

## Case presentation

**Figure 1:**
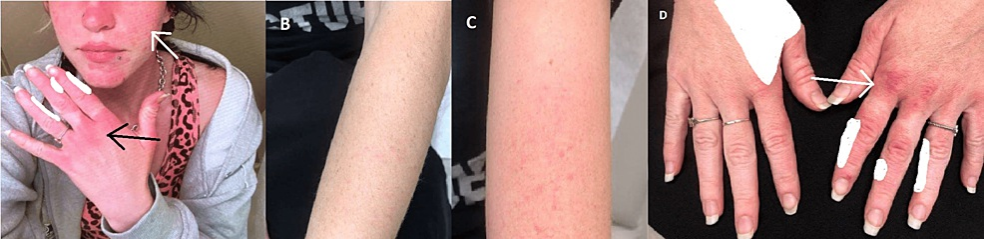
Characteristic rashes of DM. A, erythematosus butterfly rash on her face (white arrow), erythematous papular rash on her hand (black arrow). B, erythematous rash on her forearms. C, erythematous rash on her arms. D, Gottron sign, with erythematous papules over the metacarpophalangeal (white arrow) and interphalangeal joints

**Figure 2:**
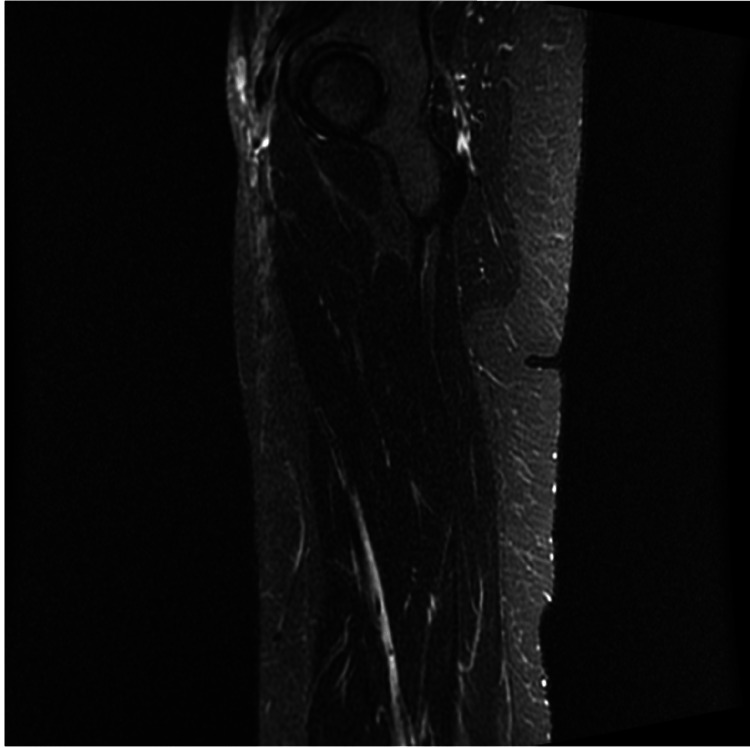
MRI of the left thigh. A, coronal T2-weighted (short tau inversion recovery) imaging showing normal muscle mass with no atrophy or edema

A 30-year-old female presented with burning, blistering, painful rashes on her arms, chest, and face, worsened by sun exposure for the past year. On examination, she had scalp tenderness and myalgia and arthralgia upper extremities. Her skin findings included an erythematosus butterfly rash on her face, an erythematous papular rash on the extensor surface of her hands, and an erythematous rash on her arms and chest (Figure 1). Neurological examination was normal, and investigations including EMG/NCV, MRI of the left thigh (Figure 2), autoimmune panel, and extended muscle-specific myositis panel including MDA5 were all negative, except for reduced C3 and C4 levels. A skin punch biopsy was done and showed compact orthokeratosis, atrophy of the epidermis, interface changes, and increased dermal mucin. The patient was started on a multidrug regimen, including methotrexate, hydroxychloroquine, and daily prednisone, which led to reductions in overall skin manifestation flare-ups and absence of muscle involvement after one year of follow-up. Intravenous immune globulin (IVIG) treatment was aborted due to significant side effects. Screening for ILD was negative. The patient was monitored by multiple specialties, including neurology, rheumatology, and dermatology. Within one year’s follow-up while on disease-modifying therapy, she was found to have reductions in overall skin manifestation flare-ups and continued absence of muscle involvement.

## Discussion

This case highlights a rare disease that can be overlooked and thus not appropriately managed. Our patient presented with predominantly skin manifestations without neurological manifestation. Given the distribution pattern of these skin findings, DM should be part of the differential diagnoses. Extensive workup from neurological examination to muscle imaging to muscle-specific enzyme testing found no muscle involvement, which favors CADM in the setting of cutaneous pathology. CADM is a rarer subtype of DM that represents approximately 20% of all cases [9]. Furthermore, we present a case of seronegative CADM as MSA, including those associated with DM, were negative. The prevalence of seronegative CADM is not established. To our knowledge, there are limited case reports on this very rare entity [9].

DM is an uncommon autoimmune disorder that primarily affects the muscles and skin. The underlying pathophysiological mechanisms of DM remain poorly understood; however, the disease is believed to arise from an aberrant immune response, which leads to inflammation and vascular damage in the affected tissues. DM can manifest in various subtypes, including classic DM, ADM, and CADM. The classic subtype is characterized by characteristic skin changes, muscle weakness, and pain, while ADM presents without muscle involvement. CADM is a rare subtype that primarily presents with skin manifestations but without muscle involvement [10]. The diagnostic criteria for DM involve typical skin alterations, muscle weakness and pain, and elevated levels of muscle enzymes in blood tests. A biopsy of affected skin or muscle tissue may also be used to confirm the diagnosis [11,12].

The incidence and prevalence of CADM are not well established due to its rarity, and it is more commonly reported in Asian populations. The natural history of CADM is not fully understood, but it is generally considered to have a milder disease course compared to classic DM, which may involve more severe muscle involvement and poorer outcomes.

DM is associated with significant extramuscular manifestations, including lung disease, cardiac abnormalities, and malignancy. ILD is an associated risk that can be progressive and fatal if not identified and treated appropriately [11]. In particular, ADM has been found to be closely associated with the development of ILD and warrants prompt screening and continued monitoring of lung disease. The incidence of ILD in patients with ADM has been shown to range from 5% to 65% [13]. Diagnosis and monitoring of ILD depend heavily on the use of CT, which shows characteristic ground glass opacities, subpleural patchy consolidation with reticulations, and bronchiectasis [14]. Anti-MDA5 positive ADM is associated with progressive ILD on CT imaging. Studies have shown that >50% of patients with rapidly progressive ILD with ADM die of respiratory failure within one year [13]. Malignancy is seen in approximately 20% of all cases of DM [5]. ADM is strongly related to the development of malignancies, with lung cancer being the most common type [15]. Other malignancies involving the breast, nasopharyngeal, genitourinary, and colon have been found in patients with ADM. Screening for malignancies is a critical component in the management of DM.

**Figure 3:**
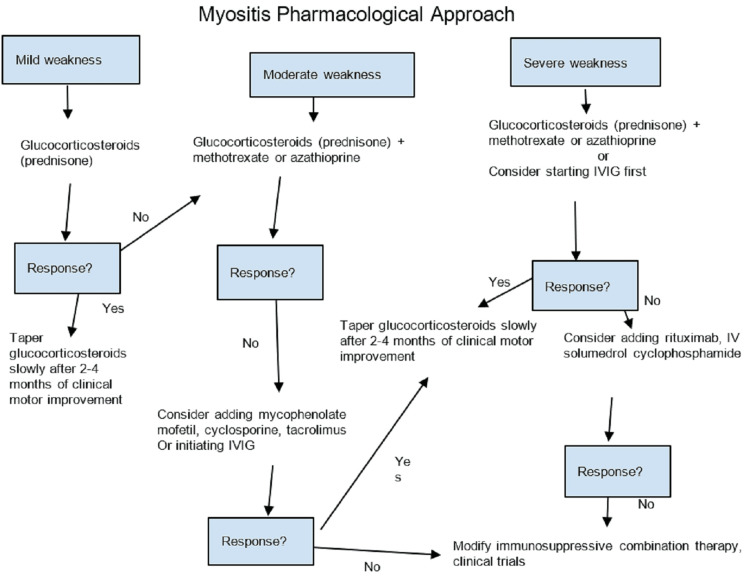
Stepwise approach to medical treatment of myositis based on clinical weakness (original media) IVIG: intravenous immunoglobulin [19]

Due to these substantial risks of potentially fatal complications, properly diagnosing and treating DM, especially with the CADM subtype, is of critical importance. Workup includes a thorough examination, muscle biopsy, muscle imaging, muscle-specific enzymes, and muscle-specific antibody testing. Muscle-specific antibodies have allowed for better prognostic capability and a more targeted therapeutic approach. Table 1 shows the common clinical presentation and degree of associated malignancy risk with MSA relating to DM. Treatment of DM is focused on immunosuppressive therapy given the pathogenesis of DM [16,17]. Though not well understood, inflammatory infiltrate consisting of CD4+ T cells, B cells, and dendritic cells occurs in muscle tissue. This inflammatory process leads to damage to muscle tissue and blood vessels. Cytokines involvement, including interferons, has also been implicated in the inflammatory process. Systemic corticosteroids, such as prednisone, have commonly been used as first-line therapy in targeting the inflammatory process. Figure 3 depicts the pharmacological approach to myositis, including DM. Other immunosuppressive agents used include methotrexate, azathioprine, mycophenolate mofetil, and IVIG [17]. The use of immunosuppressive therapeutic agents, either in monotherapy or in combination, can be tailored based on the MSA subtype. Management of DM, especially for dermatological manifestations, requires long-term immunosuppressive therapy [18].

**Table 1:** MSA by myositis subtype and classic clinical features (original table) DM: dermatomyositis, ILD: interstitial lung disease, MSA: myositis-specific antibodies [19]

MSA for DM	Clinical features
Anti-Mi-2	Classical skin manifestations of DM, moderate muscle involvement
Anti-MDA-5	Severe skin manifestations, no/little muscle involvement, associated with rapidly progressive ILD
Anti-NXP-2	Classic skin manifestations of DM, mild to moderate muscle involvement, increased risk of malignancy
Anti-SAE	Classic skin manifestations of DM, mild muscle involvement
Anti-TIF-1Y	Severe skin manifestations of DM, variable muscle involvement, high degree of associated malignancy

## Conclusions

We present a very rare case of SADM in a patient who presented with a painfully recurring rash found in a distribution pattern typical for DM. This case highlights the need for physicians to be aware of DM in making differential diagnoses for patients presenting with cutaneous symptoms. Clinical history and clinical examination are of critical importance in diagnosing DM, and workup should be initiated when clinically suspected. Muscle manifestations can lag behind the cutaneous manifestations, which can delay critical evaluation of extramuscular manifestations including ILD and malignancy which are associated with increased mortality. A multidisciplinary approach involving neurology, rheumatology, and dermatology plays a pivotal role in managing these patients from both a quality-of-life perspective, control of symptoms, and progression of the disease. MSA has become an increasingly vital component of the workup and has led to better prognostication of the severity of disease and expected complications, as well as allowed for a more targeted immunosuppressive therapeutic approach. Patients with DM, especially CADM, require close monitoring of neurological manifestations and the development of associated complications such as ILD and malignancy.
